# Supplementary material for: The Anticancer Drug Daunomycin Directly Affects Gene Expression and DNA Structure
Source: Int J Mol Sci. 2023 Apr 1;24(7):6631. doi: 10.3390/ijms24076631 (PMC10095590; doi:10.3390/ijms24076631)
Supplement: Supplementary file 1 [file ijms-24-06631-s001.zip › ijms-2255645-supplementary.pdf]

Supplementary Material

The Anticancer Drug Daunomycin Directly Affects Gene Expression and DNA Structure

Takashi Nishio [1,2], Yohji Shimada [1], Yuko Yoshikawa [1], Takahiro Kenmotsu [1], Helmut Schiessel [2] and Kenichi Yoshikawa [1,3,*]

[1] Faculty of Life and Medical Sciences, Doshisha University, Kyoto 610-0394, Japan; takashinishio7329@gmail.com (T.N.); ydu.schalke402@gmail.com (Y.S.); yoshi2989r@gmail.com (Y.Y.); tkenmots@mail.doshisha.ac.jp (T.K.)
[2] Cluster of Excellence Physics of Life, TU Dresden, 01307 Dresden, Germany; helmut.schiessel@tu-dresden.de
[3] Center for Integrative Medicine and Physics, Institute for Advanced Study, Kyoto University, Kyoto 606-8501, Japan
* Correspondence: keyoshik@mail.doshisha.ac.jp

Method for single molecular observations of T4 GT7 DNA by fluorescence microscopy (FM). Individual DNA molecules (large DNA, T4 GT7 DNA (166 kbp)) were visualized in solution by FM, as described previously [25,26,30]. DNA was dissolved in 10 mM Tris-HCl buffer at pH 7.5 with 4% (*v*/*v*) 2-ME. To observe the effects of spermine (SP) on DNA conformation, the desired concentrations of SP were added to the sample solutions. Measurements were conducted using a low DNA concentration (0.1 μM in nucleotide units) stained with YOYO-1 (0.05 μM). Single-molecule observations were performed with an inverted fluorescence microscope (Axiovert 200, Carl Zeiss, Oberkochen, Germany) equipped with a 100× oil-immersion objective lens. Fluorescent illumination was performed using a mercury lamp (100 W) via a filter set (Zeiss-10, excitation BP 450–490; beam splitter FT 510; emission BP 515–565). Images were recorded onto a DVD at 30 frames per second through a high-sensitivity EBCCD (Electron Bombarded Charge-Coupled Device) camera (Hamamatsu Photonics, Shizuoka, Japan) and analyzed with the image-processing software ImageJ (National Institute of Mental Health, MD, USA). Based on the observations of time-successive images, the probability distribution of the long-axis length of DNA in solution was evaluated. 50 DNA molecules were measured for each experimental condition.

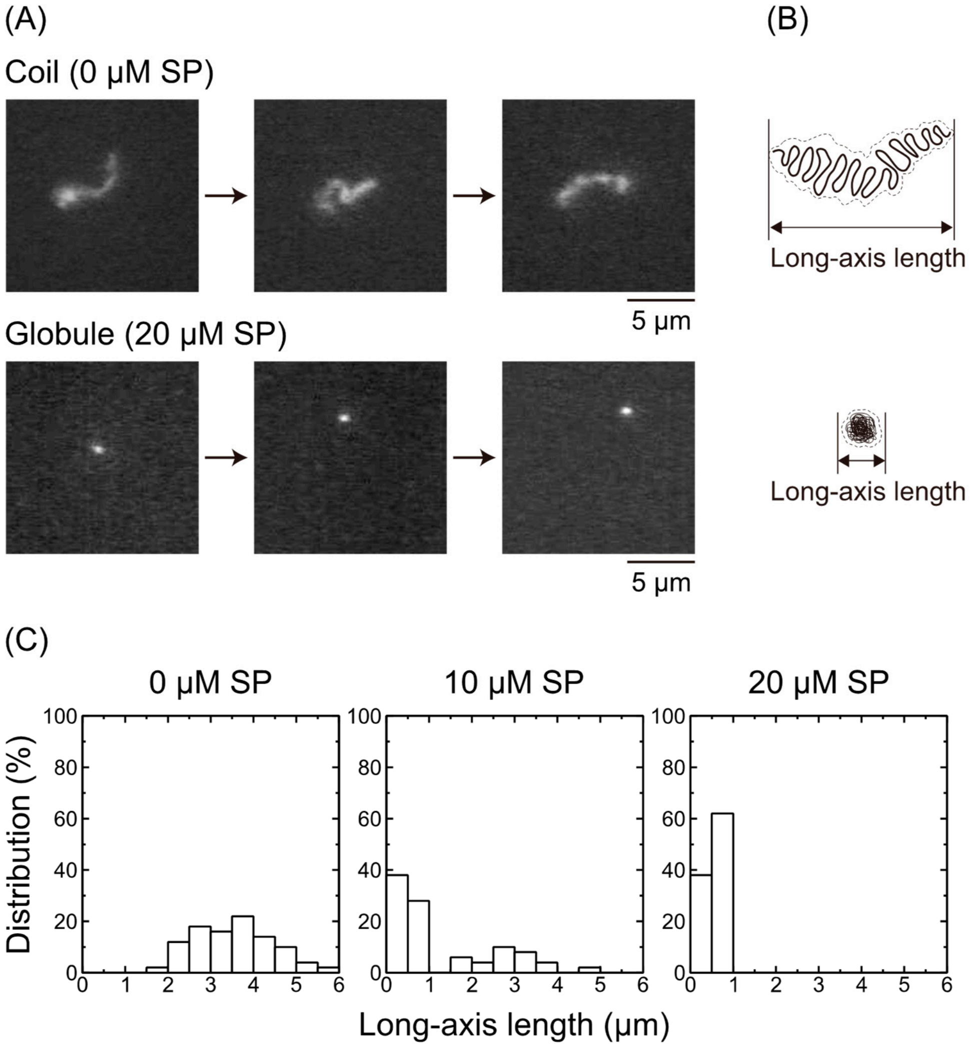

Figure S1. Effects of spermine (SP) on the long-axis length. (**A**) Typical fluorescence microscopy (FM) images of a single T4 GT7 DNA molecule in bulk solution (the total observation time is 1 sec), the top panel shows a coil in the absence of SP and the bottom panel shows a globule in the presence of SP. (**B**) Cartoon depiction of a long-axis length correlating to coil (top) and globule (bottom) DNA in (**A**). (**C**) Distribution of the long-axis length of T4 GT7 DNA in solution at different concentrations of SP. The DNA concentration was fixed at 0.1 μM in nucleotide units.
